# Xanthophylls Modulate Palmitoylation of Mammalian β-Carotene Oxygenase 2

**DOI:** 10.3390/antiox10030413

**Published:** 2021-03-09

**Authors:** Sheetal Uppal, Sergey A. Dergunov, Weiyu Zhang, Susan Gentleman, T. Michael Redmond, Eugene Pinkhassik, Eugenia Poliakov

**Affiliations:** 1 Laboratory of Retinal Cell & Molecular Biology, National Eye Institute, National Institutes of Health, Bethesda, MD 20892, USA; sheetal.uppal2@nih.gov (S.U.); sbgman@verizon.net (S.G.); 2 Department of Chemistry, University of Connecticut, Storrs, CT 06269, USA; sergey.dergunov@uconn.edu (S.A.D.); weiyu.zhang@uconn.edu (W.Z.)

**Keywords:** BCO2, palmitoylation, xanthophylls, large unilamellar vesicles, β-carotene

## Abstract

An extensive body of work has documented the antioxidant role of xanthophylls (lutein and zeaxanthin) in human health and specifically how they provide photoprotection in human vision. More recently, evidence is emerging for the transcriptional regulation of antioxidant response by lutein/lutein cleavage products, similar to the role of β-carotene cleavage products in the modulation of retinoic acid receptors. Supplementation with xanthophylls also provides additional benefits for the prevention of age-related macular degeneration (AMD) and attenuation of Alzheimer’s disease symptoms. Mammalian β-carotene oxygenase 2 (BCO2) asymmetrically cleaves xanthophylls as well as β-carotene in vitro. We recently demonstrated that mouse BCO2 (mBCO2) is a functionally palmitoylated enzyme and that it loses palmitoylation when cells are treated with β-carotene. The mouse enzyme is the easiest model to study mammalian BCO2 because it has only one isoform, unlike human BCO2 with several major isoforms with various properties. Here, we used the same acyl-RAC methodology and confocal microscopy to elucidate palmitoylation and localization status of mBCO2 in the presence of xanthophylls. We created large unilamellar vesicle-based nanocarriers for the successful delivery of xanthophylls into cells. We demonstrate here that, upon treatment with low micromolar concentration of lutein (0.15 µM), mBCO2 is depalmitoylated and shows partial nuclear localization (38.00 ± 0.04%), while treatment with zeaxanthin (0.45 µM) and violaxanthin (0.6 µM) induces depalmitoylation and protein translocation from mitochondria to a lesser degree (20.00 ± 0.01% and 35.00 ± 0.02%, respectively). Such a difference in the behavior of mBCO2 toward various xanthophylls and its translocation into the nucleus in the presence of various xanthophylls suggests a possible mechanism for transport of lutein/lutein cleavage products to the nucleus to affect transcriptional regulation.

## 1. Introduction

Most consideration of the antioxidant function of xanthophylls, polar hydroxy carotenoids, has focused on their chemical role in quenching excited triplet states of singlet oxygen by virtue of their extended conjugated bond systems. However, there is emerging evidence for a role of xanthophyll/xanthophyll cleavage products in modulating transcriptional regulation of antioxidant gene pathways.

In respect of their best-known role, high dietary intake of xanthophylls may offer protection against age-related macular degeneration (AMD), cancer and neurodegenerative diseases [1,2]. While xanthophylls account for less than 20% of the total carotenoids in the human diet, in the blood plasma the amount of xanthophylls increases to about 40% and is increased even more in the brain and retina [3,4]. Thus, xanthophylls account for about 70% of total carotenoids in all brain regions. Xanthophylls are selectively concentrated in the most vulnerable regions of polyunsaturated lipid-enriched membranes, such as in the retinal photoreceptor outer segments [5]. This localization is ideal for macular xanthophylls to act as lipid-soluble antioxidants, which is the most likely mechanism of protection against photooxidation [6]. Additionally, the high membrane solubility and preferential transmembrane orientation of macular xanthophylls [6,7] enhance their chemical and physical stability in retina and brain membranes [8] and maximize their protective action against oxidative stress in these organs [9]. Xanthophylls are capable of quenching excited triplet states of potent singlet oxygen photosensitizers. Free all-*trans*-retinal may absorb light and transfer energy from its excited triplet state to molecular oxygen, generating singlet oxygen [10]. It is postulated that the close proximity of xanthophylls allows effective energy transfer from excited all-*trans*-retinal to xanthophyll and prevents singlet oxygen generation by this photosensitizer [11]. By this mechanism, the largest part of excess energy can be transferred from potentially harmful triplets of photosensitizers to xanthophylls and dissipated as heat. The ratio of zeaxanthin (and *meso*-zeaxanthin) to lutein is higher in the macula where the strongest light is received compared to peripheral low-light-vision regions of the eye [3]. A portion of dietary lutein is converted to *meso*-zeaxanthin, a stereoisomer of zeaxanthin, presumably in retinal pigment epithelium (RPE) by RPE65 isomerase [12]. This preference for zeaxanthin has been suggested to be due to a greater antioxidant capacity, possibly due to the longer system of conjugated double bonds and membrane-stabilizing function of zeaxanthin (and *meso*-zeaxanthin) compared to lutein [12,13]. Xanthophylls are delivered to the retina with the help of high-density lipoprotein (HDL) [14] and xanthophyll-binding proteins [15]. Xanthophyll-binding proteins have been described for both zeaxanthin and lutein [16]. However, the role of β-carotene oxygenase 2 (BCO2), an enzyme that can cleave xanthophylls [17], is not thoroughly studied.

BCO2 is present in the brain, retina and RPE; everywhere xanthophylls accumulate. BCO2 is well characterized as a carotenoid metabolizing enzyme [18,19,20,21] that is widely distributed in tissues, including the retina, RPE, skeletal muscle, small intestine and liver [22]. A deficiency of BCO2 was found to be associated with accumulation of carotenoids in the adipose tissues [23], such as subcutaneous adipose tissue. This leads to the occurrence of yellow fat in sheep [24], cow [25], and yellow skin in chicken [26]. The abSNP rs2250417 in BCO2 has one of the strongest instances of statistical significance for association with AMD of carotenoid metabolism genes [27]. The two minor alleles for SNP rs2250417 in BCO2 account for an increase in risk for AMD by almost 50% [27]. Additionally, BCO2 deficiency in mice leads to stimulation of oxidative stress and inflammation in hypothalamic tissues on a low carotenoid diet [28].

To better study the role of BCO2 in metabolism of xanthophylls, we developed xanthophyll-containing large unilamellar vesicles (LUVs) to efficiently deliver xanthophylls to cells. We chose to express mBCO2 in human cell culture because it has only one major isoform while human and monkey BCO2 have several major isoforms. Only one of these has a N-terminal mitochondrial signal and it is still hotly debated if this isoform manifests as an active BCO2 enzyme [17,29,30,31]. Here, using LUVs, we describe that lutein modulates the palmitoylation status of mouse BCO2 (mBCO2) and changes its cellular localization in HEK293F cells from mitochondria to nucleus upon binding. We have detected the same effect for zeaxanthin and violaxanthin but to a lesser degree and our modeling analysis suggests a possible explanation of the difference. Accumulation in the nucleus of mBCO2 loaded with xanthophylls could directly affect gene expression and may provide a mechanism whereby xanthophylls/xanthophyll cleavage products could elicit a transcriptional response to oxidant stress.

## 2. Materials and Methods

### 2.1. Materials

1,2-dimyristoyl-sn-glycero-3-phosphocholine (DMPC), 1,2-dimyristoyl-sn-glycero-3-phospho-L-serine (sodium salt) (DMPS), 1,2-dipalmitoyl-sn-glycero-3-phosphocholine (DPPC), 1,2-dipalmitoyl-sn-glycero-3-phospho-L-serine (sodium salt) (DPPS) were purchased from Avanti Polar Lipids, Inc. (Alabaster, AL, USA). (R)-(+)-limonene and tricine were used as received (Sigma-Aldrich, St. Louis, MO, USA). Nitrogen-purged hexane, dichloromethane (DCM) and chloroform (CHCl_3_) were passed through an activated alumina column, dried with CaSO_4_, and stored over 4Å molecular sieves. All kinetic experiments were performed with the same batch of samples. Lutein and violaxanthin were purchased from Cayman Chemicals (Ann Arbor, MI, USA), and zeaxanthin (65%) from Toronto Research Chemicals (Toronto, ON, Canada).

### 2.2. Preparation of Large Unilamellar Vesicles (LUVs) with Xanthophylls

Aqueous lipid dispersions of LUVs were prepared by first mixing the required amounts of lipids, carotenoids, and limonene (used here for better solubilization of carotenoids in the lipid bilayer) in chloroform. All samples were prepared using similar protocol to the one described previously [32]. Amounts of lipids were the same, 10 mg/mL, limonene: lipid = 1:1 (mol/mol), while amounts of carotenoids were different: 0.5 mg zeaxanthin in 1.5 mL of buffer (using DPPC/DPPS or DMPC/DMPS lipids); the lutein preparation contained 0.25 mg lutein in 1.5 mL of buffer (using DMPC/DMPS lipids); and the violaxanthin preparation contained 0.25 mg violaxanthin in 1.5 mL of buffer (using DMPC/DMPS lipids). All samples were prepared with a mole fraction of 7% PS (*sn*-glycero-3-phospho-L-serine) to avoid multilamellar stack formation [33]. The solvent was evaporated with an inert gas stream to constant weight. The lipid film was hydrated with 100 mM tricine-KOH pH 8.0 buffer (prepared with H_2_O) at room temperature with intermittent gentle vortex mixing. The lipid suspension was passed through 0.4, 0.2, 0.1 μm Nucleopore polycarbonate membranes (21 times through each membrane) using a mini-extruder (Avanti Polar Lipids). The LUVs were mixed with hexane and shaken gently for 20 min to remove limonene as confirmed by GC-MS. The LUVs were then decanted from the organic layer and the aqueous solution was purged with nitrogen and degassed to remove traces of hexane.

### 2.3. Generation of Expression Vectors

Mouse BCO2 cDNAs were prepared as described [34] and used to generate vectors for the expression of BCO2 in mammalian cells. Briefly, BCO2 was subcloned into the bicistronic expression vector pVitro2 (InvivoGen, San Diego, CA, USA) and into the Gateway cloning vector pcDNA6.2c-Lumio-DEST vector (Thermo Fisher (Invitrogen), Carlsbad, CA, USA) to generate untagged or C-terminal V5/lumio tagged versions of mouse BCO2, respectively. All constructs and mutants were sequenced to verify the orientation and accuracy of the ORFs and/or the changes introduced.

### 2.4. Cell Culture

Human 293F FreeStyle (Thermo Fisher, Invitrogen) suspension cells were grown in serum-free FreeStyle 293 expression medium (Invitrogen) and transfected according to the previously published protocol [35]. Briefly, a typical transfection experiment used 3 × 10^7^ cells in 28 mL of FreeStyle medium mixed with 2 mL of OptiMem-I reduced serum medium containing 40 μL 293fectin transfection reagent (Invitrogen) and 20 μg of each expression plasmid under study. Cells were grown with shaking at 125 rpm on an orbital shaker platform in a 37 °C incubator with a humidified atmosphere of 8% CO_2_ for 48 h total. In total, 200 µL of vesicles with xanthophylls were added, incubating for 5 hr under standard growth conditions.

### 2.5. Antibodies

Rabbit polyclonal antibody 186 was custom made against the mouse BCO2 multiple antigenic peptides (MAP)-SKFLQSDTYKANSAG peptide and 7055 rabbit polyclonal antibody was produced by co-immunization of the two human BCO2 MAP-SHENLHQEDLEKEGGIE and MAP-QDNGRTLEVYQLQNLRKAG peptides.

### 2.6. Characterization of Large Unilamellar Vesicles (LUVs) with Xanthophylls

#### 2.6.1. Dynamic Light Scattering (DLS)

The hydrodynamic diameter and polydispersity index (PDI) were measured at 30 °C on a Malvern Nano-ZS zetasizer (Malvern Instruments Ltd., Worcestershire, UK) equipped with a 4 mW helium-neon laser operated at 633 nm with a fixed scattering angle of 173°. An 80 μL sample was placed into disposable cuvettes without dilution (70 μL, center height 8.5 mm, BRAND^®^ UV-Cuvette micro). Data were processed using non-negative least squares (NNLS) analysis.

#### 2.6.2. GC–MS Analysis

A Shimadzu GC-2010 Plus system with an AOC-20i Auto-Injector and a GCMS-QP2010 SE ion trap MS system (Shimadzu, Kyoto, Japan) was used for GC-MS analysis using the electron impact ionization mode. Chromatographic separations were performed on a Shimadzu SH-Rxi-5SiL MS capillary column (30 m × 0.25 mm, 0.25mm film thickness; non-polar phase: Crossbond™ 100% dimethyl polysiloxane as stationary phase). The temperatures of the injector and the GC-MS transfer line were 170 and 280 °C, respectively. The carrier gas was ultrahigh purity helium (Airgas); the flow rate was 1.0 mL/min. The mass spectrometer was operated using the following parameters: the ratio of the split injection was 20:1, ionization voltage was 70 eV; ion source temperature was 200 °C; scan mode, 30.0–500.0 (mass range); scan rate, 5000 amu/s, and 3.68 scans/s; start time was 2 min. Electron multiplier (EM) voltage was obtained from autotune. The oven temperature was programmed to hold at 60 °C for 2 min, increase to 300 °C at 50 °C/min, and hold at 300 °C for 3 min.

To measure the residual limonene in LUVs, a 100 μL aliquot is mixed with 1.9 mL of hexane and 50 mg of CaSO_4_ (used here to disrupt the LUVs) and stirred for 30 min to extract limonene. The data were averaged from at least three independent measurements.

#### 2.6.3. Small-Angle X-ray Scattering (SAXS)

The structural characteristics of LUVs were studied using small-angle X-ray scattering (SAXS). SAXS patterns were obtained using a Bruker NanoStar instrument equipped with a turbo rotating anode operated at 50 kV and 50 mA, evacuated beam path, two-pinhole collimators, Göbel mirrors selecting Cu-Kα radiation, and a large 2D Vantec-2000 detector. Samples were measured in 1.5 mm quartz capillaries; the measurement time was 3 h. Scattering patterns were collected in the range 0.006 Å-1 < q < 0.35 Å^−1^. The sample to detector distance of 67.8 cm was verified using silver behenate as a calibration standard. The SAXS patterns were corrected for sample transmission and empty cell scattering. One-dimensional (1D) SAXS patterns were obtained by azimuthal integration of the resulting 2D images around the beam center, to obtain the intensity (ISAXS) vs. q profiles. The magnitude of the scattering vector was calculated as q = (4π/λ) sin(°/2), where θ is the scattering angle and λ is the X-ray wavelength for Cu-Kα (λ = 1.5418 Å).

#### 2.6.4. Spectroscopic Determination of the Concentration of Xanthophylls

The concentrations of xanthophylls were measured using a 2 mm optical path quartz cell in an Agilent Cary 60 UV-Vis spectrophotometer. To measure the concentration of xanthophylls in LUVs, a 10 μL aliquot is mixed with 300 μL of DMF. Standards and samples were measured at least 3 times and the data were averaged. Final concentrations of xanthophylls in LUVs measured by UV/Vis: zeaxanthin 0.038 g/L (DPPC/DPPS, 0.45 µM final), zeaxanthin 0.14 g/L (DMPC/DMPS); lutein 0.0125 g/L (DMPC/DMPS, 0.15 µM and 0.06 µM final); violaxanthin 0.07 g/L (DMPC/DMPS, 0.6 µM final).

### 2.7. BCO2 Protein Palmitoylation Was Analyzed by Acyl-Resin-Assisted Capture (Acyl-RAC)

For cell lysis and the Acyl-RAC assay, we followed the same protocol as previously described in detail, with slight modifications [34,36]. BCO2-overexpressing HEK293F cells were washed with 1X-PBS and resuspended in lysis buffer (50 mM HEPES (pH 7.4) containing 150 mM NaCl, 5 mM EDTA, 1% glycerol, and 1X complete protease inhibitor cocktail (Roche Diagnostics)). Resuspended cells were lysed using N_2_ cavitation followed by centrifugation at 900× *g* for 10 min at 4 °C to remove the cell debris and nuclei. The clarified supernatant was then subjected to centrifugation at 20,000× *g* for 30 min at 4 °C to obtain heavy membrane (mitochondrial) pellet and post-mitochondrial supernatant (light membrane and cytosol) fractions. Typically, a 1000 µg amount of resuspended heavy membrane pellet protein (resuspended in lysis buffer containing 0.1% Triton X-100) was used for palmitoylation detection assay. Briefly, free cysteine sulfhydryl (-SH) groups were blocked with 0.5% (*v/v*) S-methyl methanethiosulfonate (MMTS) containing blocking buffer for 15 min at 40 °C. The blocked protein samples were then subjected to acetone protein precipitation. The protein pellet was then resuspended in 550 µL 100 mM HEPES containing 5 mM EDTA and 1% SDS (*v/v*). The samples were then divided into two 250 µL aliquots (containing 400 µg protein amount) and the remaining 50 µL was used as an input. The samples were treated with 250 mM hydroxylamine (NH_2_OH, HAM) or control (250 mM NaCl). To capture proteins with free -SH groups, each sample was mixed with 10 mg activated thiol-sepharose 4B beads (Sigma) and incubated for 2 h at room temperature with continuous end-over-end rotation. After incubation, the beads were washed, and bound proteins were eluted by boiling the beads with an aliquot of 50 µL elution sample buffer. The input and eluted fractions from “HAM” and “control” samples were separated by SDS-PAGE and analyzed by Western blotting.

### 2.8. Immunocolocalization Studies BCO2 Protein in Different Organelles upon Substrate Treatment Using Confocal Microscopy

BCO2-overexpressing COS7 cells (1 × 10^6^ cells/mL) seeded on poly L-lysine coated 18 mm coverslips were analyzed by immunofluorescence microscopy to determine the localization of BCO2 protein in different subcellular organelles as described previously [34]. Briefly, COS7 cells were transfected with 20 µg of BCO2-Lumio V5 tag plasmid using Fugene^®^ 6 transfection reagent (1:6 DNA: Fugene 6 ratio). After 43 h, BCO2-transfected cells were treated with substrate-encapsulated LUVs for 5 h. Fixed cells were immunostained with V5 monoclonal and polyclonal antibodies alone and together with primary antibodies specific for different subcellular organelles (ER (PDIA3), Golgi (MAN2A1), mitochondria (COX IV and HSP60), and peroxisomes (PMP70)), followed by Alexa fluorophore-conjugated secondary antibodies (Invitrogen). Cell nuclei were stained with DAPI (1 µg/µL solution; Sigma). Slides were then visualized with a Zeiss LSM 700 confocal microscope using a 40X oil immersion lens/1.4-NA and Zeiss ZEN software. Pearson’s correlation coefficient values were determined for analysis of the co-localization of BCO2 protein and different organelles and represented as mean ± standard deviation. For nuclear co-localization of BCO2 protein, we measured the Pearson’s correlation coefficient of BCO2 with DAPI-stained nuclei using the results from three independent experiments: single labeling of BCO2 “alone”, double labeling of BCO2 with mitochondrial markers COX IV and HSP60. Results shown were typical of a minimum of three independent experiments with 5–10 fields of view containing on average 1–10 cells/field of view. For quantification and statistical analyses, at least 100 cells were observed for each organelle and nuclear co-localization.

### 2.9. Construction of Models and Ligand Docking Simulations

A model of mBCO2 was constructed with the Swiss-Model program using the RPE65 4F30 crystal as a template [37]. The loop carrying the PDPCK motif is unresolved in this crystal and is given in this model as a random loop. To model the unresolved areas, the mBCO2 sequence with distances specified for the catalytic histidines was submitted to the I-Tasser server [38,39,40]. Five models were obtained; however, the side chains of the Fe-coordinating histidines and glutamates were displaced relative to both the Swiss-Model and RPE65 crystals. The sidechains of the histidines and glutamates of the top I-Tasser model were modified based on the Swiss-Model and the RPE65 crystal structures. Finally, to model an active state, the Fe center and the O_2_ and OH of the VP14 crystal (PDB: NPE3) were aligned with both models and integrated into the models. Clashes resulting from the introduction of O_2_ and OH on the Fe center were corrected by torsioning the relevant residues. Ligand dockings were carried out using Autodock Vina [41]. In general ligand restraints chosen allowed for all possible torsions.

## 3. Results

### 3.1. Xanthophyll Delivery System

**Figure 1:**
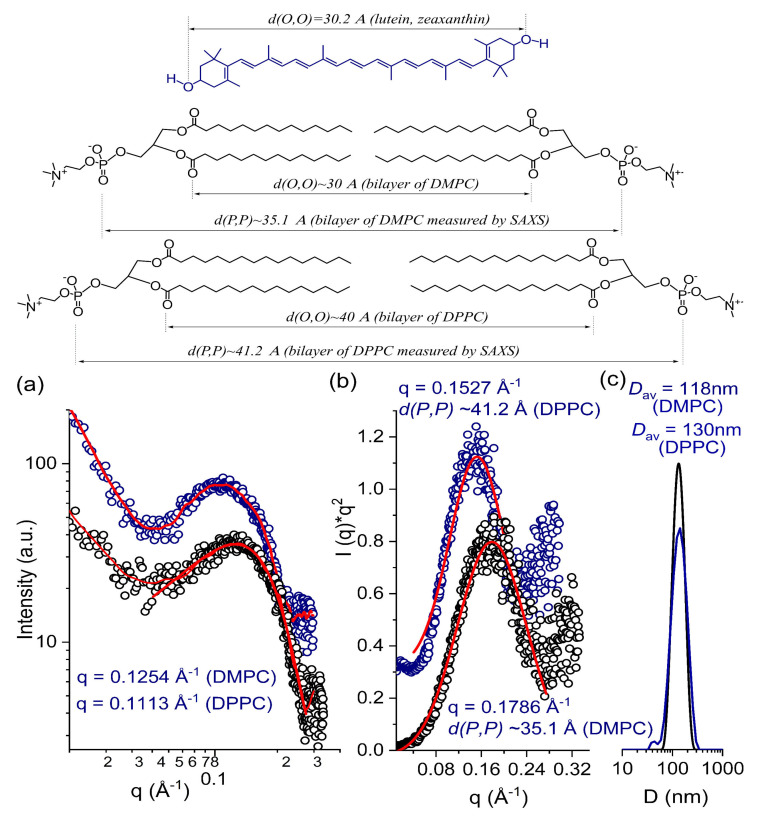
Top: Intramolecular dimensions of carotenoids and DMPC/DPPC bilayer as calculated by molecular mechanics [42,47]. The length of the carotenoid (lutein or zeaxanthin) corresponds to the length of the lipophilic segment of the DMPC phospholipid (double the average distance between the carbonyl group and the methyl group of the DMPC), and does not match the length of the lipophilic segment of the DPPC. Bottom: (**a**) SAXS data of DMPC-zeaxanthin (black) and DPPC-zeaxanthin (blue) LUVs; (**b**) Kratky plot from SAXS data, supporting the formation of unilamellar LUVs; and (**c**) Size distribution and the average diameter of DMPC-zeaxanthin (black) and DPPC-zeaxanthin (blue) LUVs measured by DLS (Figure S1).

LUVs were prepared by hydration of lipid with carotenoids and assisting limonene, followed by extrusion and extraction of limonene to yield unilamellar vesicles with a narrow size distribution, as confirmed by SAXS and DLS, respectively (Figure 1). The amount of carotenoids associated with LUVs was determined by UV-vis spectroscopy following a previously published protocol [42]. Previously, it was shown that, depending on the structure of the substrate, as well as on the composition of lipids, hydrophobic carotenoids are oriented differently in the phospholipid bilayer of delivery vesicles [43]. Thus, symmetrically oxy-functionalized carotenoids intercalate into phospholipid membranes perpendicular to the membrane surface [44], while fully non-polar β-carotene is intercalated parallel to the surface within the hydrophobic core of phospholipid bilayers [44,45]. In addition, the molecular length of a carotenoid affects the degree of its intercalation into phospholipid bilayers, depending on the membrane thickness [42]. In fact, zeaxanthin (C_40_) was better incorporated into unilamellar vesicles of dimyristoylphosphatidylcholine (n-C_14_), whereas decaprenozeaxanthin (C_50_) was better adopted in unilamellar vesicles of dipalmitoylphosphatidylcholine (n-C_16_). On the other hand, the inclusion of large hydrophobic molecules into the phospholipid bilayer can change the structure as well as the thickness of vesicles [46]. To understand the membrane structure of vesicles as well as the organization of carotenoids within DMPC and DPPC LUVs with associated zeaxanthin, X-ray scattering measurements were performed. The phosphate-phosphate (p-p) thicknesses obtained from the SAXS data fit gave values of ~35.1 Å for DMPC-zeaxanthin membranes and ~41.2 Å for DPPC-zeaxanthin membranes, which is the typical thickness for DMPC and DPPC-carotenoid LUVs [42,46]. The length values (Figure 1) of the carotenoid (lutein or zeaxanthin) correspond well to the length of the lipophilic segment of DMPC, but not DPPC. This is the reason for the much weaker incorporation of lutein (or zeaxanthin) into DPPC membranes compared to DMPC [42,47].

### 3.2. Palmitoylation of Mouse BCO2 (mBCO2) in the Presence of Xanthophylls

**Figure 2:**
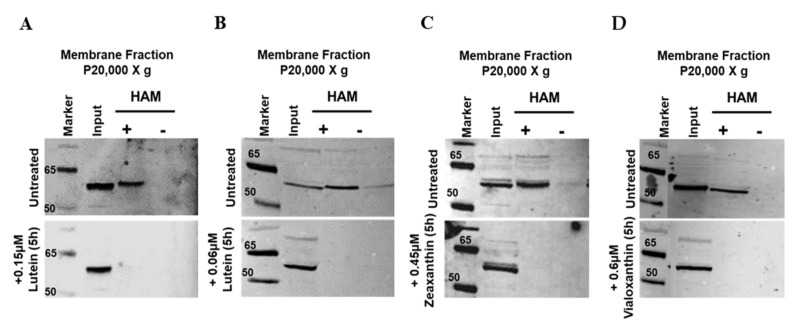
mBCO2 loses its palmitoylation in the presence of substrate. The palmitoylation status of mBCO2 protein was analyzed by Acyl-RAC assay in the presence of different substrates: (**A**) 0.15 μM lutein; (**B**) 0.06 μM lutein; (**C**) 0.45 μM zeaxanthin; and (**D**) 0.6 μM violaxanthin. Equal amounts (~50 μg) of the total (indicated as “input”) and eluted protein from control (indicated as “-”) and hydroxylamine (HAM)-treated (indicated as “+”) were subjected to SDS-PAGE and immunoblotting. Western blot results are representative of three independent experiments as shown in the Supplementary figures.

We previously established that mBCO2 in eukaryotic HEK293F cells is palmitoylated. We discovered that in the presence of the mBCO2 substrate β-carotene mBCO2 loses palmitoylation [34]. Knowing that BCO2 cleaves xanthophylls [17,48,49] we decided to run palmitoylation assays to study the palmitoylation status of mBCO2 in the presence of xanthophylls. We first tried to deliver xanthophylls with the detergent Tween 40 [50]. We found that a higher concentration of Tween 40 (0.1%) led to HEK293F cell apoptosis as was previously described for HepG2 cells [51], while a lower concentration (0.01%) eluted mBCO2 from membranes during the Acyl-RAC assay even without hydroxylamine treatment (data not shown). Therefore, we established a new method to deliver xanthophylls without detergents using unilamellar LUVs as described above. The content of xanthophylls in the HEK293F cells after 5 h of treatment was similar to a Tween 40 delivery system in ARPE-19 cells (1–2%) (Figure S2) [52]. Subsequently, we used the acyl-RAC method as described previously. The membrane fraction of HEK293F cells expressing mBCO2 was subjected to treatment with hydroxylamine (+HAM) and an equal portion of the fraction was treated with 250 mM NaCl (−HAM) which served as control. In the absence of substrates, mBCO2 protein showed an intense protein band in the HAM-treated sample (Figure 2A–D untreated panels, full Western blots are presented in Figure S3), while there was no protein band in the control NaCl-treated sample, indicating S-palmitoylation of BCO2 protein. In contrast, when cells were pre-treated with 0.15 µM lutein and 0.06 µM in DMPC/DMPS micelles, no protein band was detected in the HAM-treated sample (Figure 2A,B and Figure S3A,D, lutein panel). Similar results were obtained with DPPC/DPPS-encapsulated zeaxanthin (0.45 µM) (Figure 2C and Figure S3B) and DMPC/DMPS-encapsulated violaxanthin (0.6 µM) (Figure 2D and Figure S3C).

### 3.3. Sub-Cellular Localization of mBCO2 with and without Xanthophylls. Shuttling the Enzyme to the Nucleus

**Figure 3:**
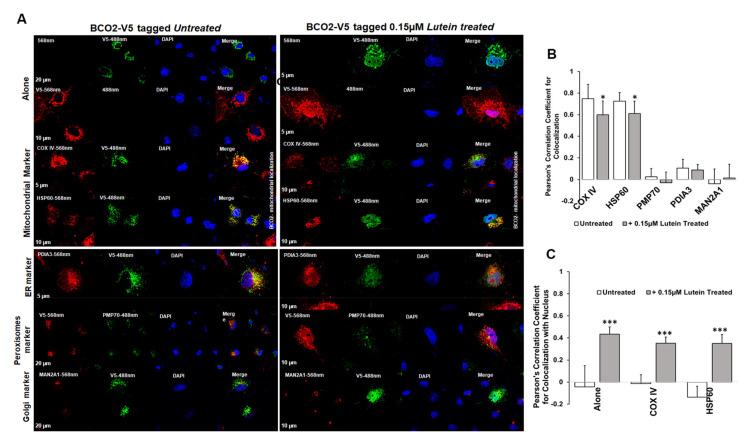
Characterization of subcellular localization of mBCO2 treated with lutein. (**A**) COS7 cells expressing mBCO2 protein treated with 0.15 μM lutein for 5 h were immunoassayed using antibodies against V5 tag and different organelle marker proteins as indicated in the Materials and Methods section. Pearson’s correlation coefficient for colocalization of V5-tagged mBCO2 protein with different organellar marker proteins (**B**) and with nucleus (**C**); * *p* ≤ 0.05; *** *p* ≤ 0.0005.

**Figure 4:**
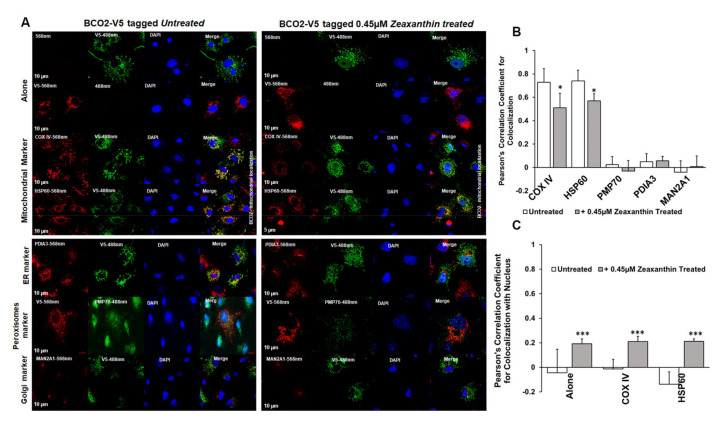
Characterization of subcellular localization of mBCO2 treated with zeaxanthin. (**A**) COS7 cells expressing V5-tagged mouse BCO2 protein treated with 0.45 μM zeaxanthin for 5 h were immunoassayed using antibodies against V5 tag and different organelle marker proteins as indicated in the Materials and Methods section. Pearson’s correlation coefficient s were calculated for colocalization of V5-tagged mBCO2 protein with different organellar marker proteins (**B**) and with nucleus (**C**); * *p* ≤ 0.05; *** *p* ≤ 0.0005.

**Figure 5:**
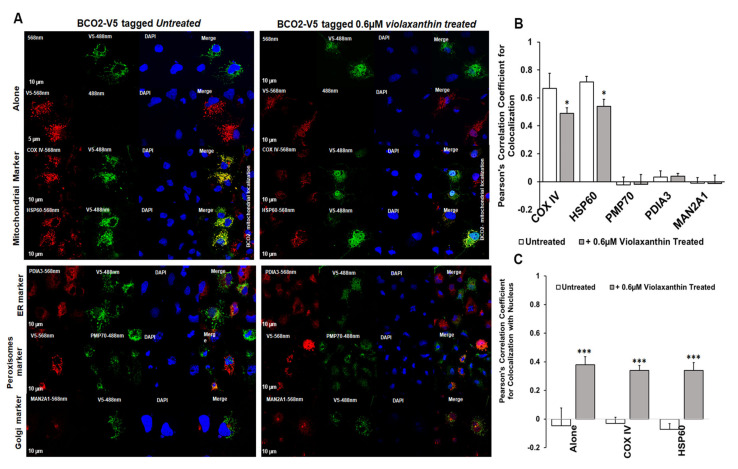
Characterization of subcellular localization of mBCO2 treated with violaxanthin. (**A**) COS7 cells expressing mBCO2 protein treated with 0.6 μM violaxanthin for 5 h were immunoassayed using antibodies against V5 tag and different organelle marker proteins as indicated in the Materials and Methods section. Pearson’s correlation coefficients were calculated for colocalization of V5-tagged mBCO2 protein with different organellar marker proteins (**B**) and with nucleus (**C**); * *p* ≤ 0.05; *** *p* ≤ 0.0005.

Next, we examined BCO2 localization by immunofluorescence microscopy using mBCO2 transfected COS7 cells with various organellar markers (for mitochondria, peroxisomes, endoplasmic reticulum (ER), and Golgi). We confirmed that V5 tag antibodies recognize specifically mBCO2-V5 protein in transfected HEK293F cells (Figure S4). For nuclear labeling, we used DAPI nuclear stain. Immunofluorescence results as shown in Figure 3A (upper left panel) revealed the extensive mitochondrial colocalization with both COXIV and heat-shock protein 60 (HSP60) (Figure 3A, upper left panel). We did not observe any colocalization of mBCO2 with other organelles (Figure 3A, bottom left panel, and 4B) as indicated by their low correlation coefficient score compared with mitochondrial localization. Our data are in full agreement with previous results for human BCO2 [21,34]. However, upon addition of substrates to mBCO2 we observed that a fraction of mBCO2 colocalizes with the nuclear DAPI stain and the colocalization with mitochondrial markers is significantly decreased (Figure 3A, top right panel, C). The percent of colocalization with nuclear marker is higher when cells are treated with lutein and violaxanthin (Figure 4A–C) than with zeaxanthin (Figure 5A–C).

**Figure 6:**
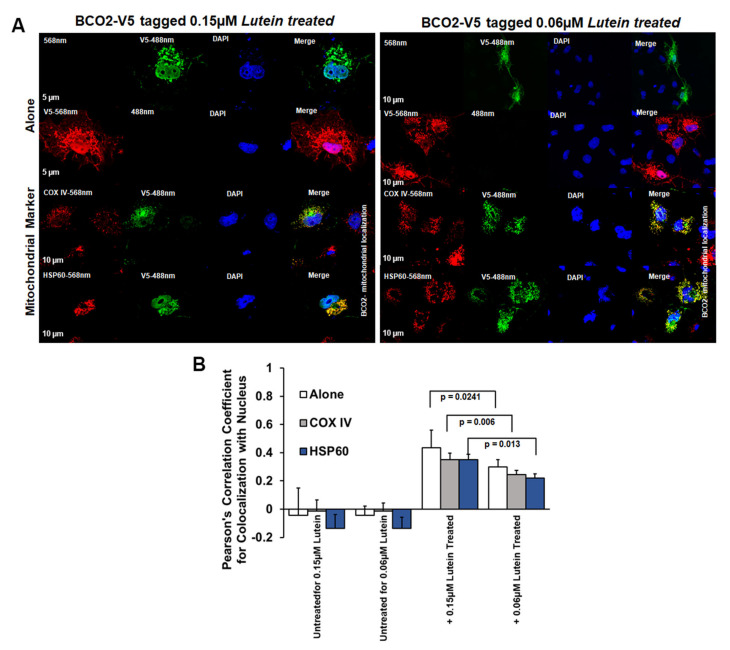
(**A**) Cellular Localization of V5-tagged mouse BCO2 with and without lutein: mBCO2 with and without 0.15 µM lutein; (left panel) mBCO2 with and without 0.06 µM lutein; (right panel). Pearson’s correlation coefficients were calculated for colocalization of V5-tagged mBCO2 protein with nucleus (**B**).

When we performed the experiment using a lower concentration of lutein (0.06 µM) we observed that the percent colocalization of BCO2 in the nucleus decreased in a lutein concentration-dependent manner (Figure 6A–C). We have previously observed mBCO2 colocalization with the nuclear DAPI stain when cells were treated with β-carotene [34]. It correlates with our observation from the acyl-RAC assay that residual mBCO2 palmitoylation is seen in some samples in the presence of zeaxanthin, violaxanthin and a low concentration of lutein (Figure S3).

### 3.4. Modeling of Xanthophyll Docking in Mouse BCO2

**Figure 7:**
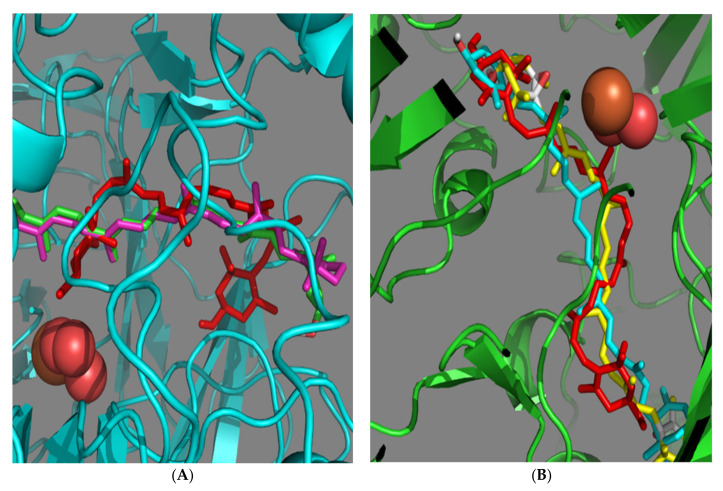
Visualization of carotenoid docking (**A**) on the mBCO2 protein I-TASSER model (lutein in green and β-carotene in purple, zeaxanthin in red and protein in cyan) and (**B**) on the mBCO2 protein Swiss-Model model (violaxanthin in yellow, lutein in cyan, zeaxanthin in red and protein in green).

To understand these findings, we performed modeling of substrate (lutein, zeaxanthin and violaxanthin) docking in mBCO2. The Swiss-Model random coil model of the bovine RPE65 4F30 crystal with the helical -PDPCK- containing loop modeled in (using I-TASSER as described in Methods) was used as a basis to model mBCO2. We observed the highest binding energy to the Swiss-Model model of mBCO2 protein with lutein and the lowest with zeaxanthin. However, zeaxanthin binding energy significantly increased in Model1 (clash-free I-TASSER model which corrected the catalytic H and E residues orientation from the Swiss-Model model) docking simulation, while lutein and violaxanthin binding energy did not change (Table 1). Additionally, zeaxanthin did not line up well in the mBCO2 active site and curled up in both of the models (Figure 7A,B).

## 4. Discussion

Here we show that xanthophylls affect the palmitoylation status of mBCO2 and direct translocation of the enzyme to the nucleus where xanthophylls could modulate gene expression and exert antioxidant properties through activation of cellular oxidative stress response genes. To accomplish this, we developed a new unique non-disruptive way for membranes to deliver xanthophylls to cells. Thus, we produced LUVs with xanthophylls by using limonene to assist solubilization of the xanthophyll in bilayers made of phospholipids of the appropriate size. Our findings expand our previous findings regarding substrate-induced depalmitoylation and organellar relocalization of mBCO2 [34] and suggest a common effect of carotenoids on this enzyme.

The catalytic activity of mBCO2 towards certain oxidative metabolites of zeaxanthin was recently documented [17]. There are numerous other xanthophylls in the human diet. For example, violaxanthin is a diepoxy derivative of zeaxanthin and accumulates in a significant amount in human ovaries [53]. However, it was not known if it is a substrate and if mBCO2 could cleave it. Our results suggest that it does. It will be useful to better understand substrate structural requirements for mBCO2 function in cells.

Xanthophylls are well known for their antioxidant protective properties which may play a role in delaying chronic diseases. Despite this, it is still far from clear how xanthophylls exert the full extent of their antioxidant properties [54]. A widely proposed pathway is that they dissipate excess energy from potentially harmful oxidants, such as excited triplet states of singlet oxygen, by virtue of their extended conjugated bond systems and thereby protect membranes from oxidant stress [11]. Recently, however, a body of literature has begun to accumulate, pointing to direct regulation of gene expression by xanthophylls [50,55]. This would be analogous to the role of β-carotene metabolites in transcriptional regulation: β-apocarotenoids have been recently found to function as transcriptional regulators, specifically as nuclear receptor antagonists, which inhibit retinoic acid activities [56,57,58,59]. In this regard, lutein has been shown to activate Nrf2, an emerging regulator of cellular resistance to oxidants, and to affect Nrf2 pathway genes in retinal cells [50,60]. It has been demonstrated that lutein effectively protects ARPE-19 from damage generated by hyperglycemia by activating Nrf2 through its regulators, suggesting a preventive role of lutein against diabetic retinopathy [60]. How lutein (and other carotenoids/carotenoid metabolites) might enter the nucleus occurs is currently unknown.

In this respect, we previously described that presence of β-carotene changes the palmitoylation status of mBCO2 and that we can see residual colocalization with the nucleus [34]. Thus, our results with lutein, zeaxanthin and violaxanthin extend these prior findings by demonstrating that, generally, mBCO2 is palmitoylated in the absence of substrates and that it loses palmitoylation when substrates are present in the cells. Therefore, it prompts us to propose that loss of palmitoylation upon substrate-treatment somehow influences the BCO2-mitochondrial localization and promotes the shuttling of BCO2 to the nucleus. The mechanism of substrate bound-BCO2 shuttling to the nucleus is still unclear and needs to be investigated in further detail to explore the unclear function of BCO2 in the nucleus. Differences in binding of substrates to mBCO2 as demonstrated in our molecular docking experiments could define differences in nucleus shuttling and antioxidant effect of the various xanthophylls. Additionally, it is important to further study if mBCO2 could work as a transporter of xanthophylls and their metabolites to the nucleus (in addition to β-carotene and its metabolites), and to elucidate the potential mode of action of xanthophylls and their enzymatic metabolites on gene expression in relation to an oxidative stress response, in addition to their quenching properties.

## Figures and Tables

**Table 1:** AutoDock Vina modeling of the affinity of carotenoids to mBCO2. The lowest (best) docking energy is used.

Carotenoid	SwissPro Model	I-TASSER Model1
violaxanthin	−11.0	−10.8
zeaxanthin	−11.8	−10.1
lutein	−10.5	−10.4
β-carotene	−11.8	−10.6

## Data Availability

The data presented in this study are available in this article and Supplementary Material here.

## Supplementary Materials

The following are available online at https://www.mdpi.com/2076-3921/10/3/413/s1, Figure S1: DLS data for LUVs with xanthophylls; Figure S2: Detection of mouse BCO2 palmitoylation by acyl-RAC assays. Raw Western blots; Figure S3: mBCO2 protein expression in the COS7 cells transfected with V5-tagged mBCO2. COS7 cells expressing mBCO2 protein treated with different substrates for 5h were grown on poly L-lysine coated coverslips and subjected to immunolocalization studies using confocal microscopy. For immunoblotting, post-nuclear supernatant was separated by SDS-PAGE. The presence of V5-tagged mBCO2 was probed by immunoblotting with rabbit polyclonal anti-mouse BCO2 (green) and mouse monoclonal anti-V5 (red) antibodies.
