# Supplementary material for: Xanthophylls Modulate Palmitoylation of Mammalian β-Carotene Oxygenase 2
Source: Antioxidants (Basel). 2021 Mar 9;10(3):413. doi: 10.3390/antiox10030413 (PMC8000801; doi:10.3390/antiox10030413)
Supplement: Supplementary file 1 [file antioxidants-10-00413-s001.pdf]

SUPPLEMENTAL FIGURES

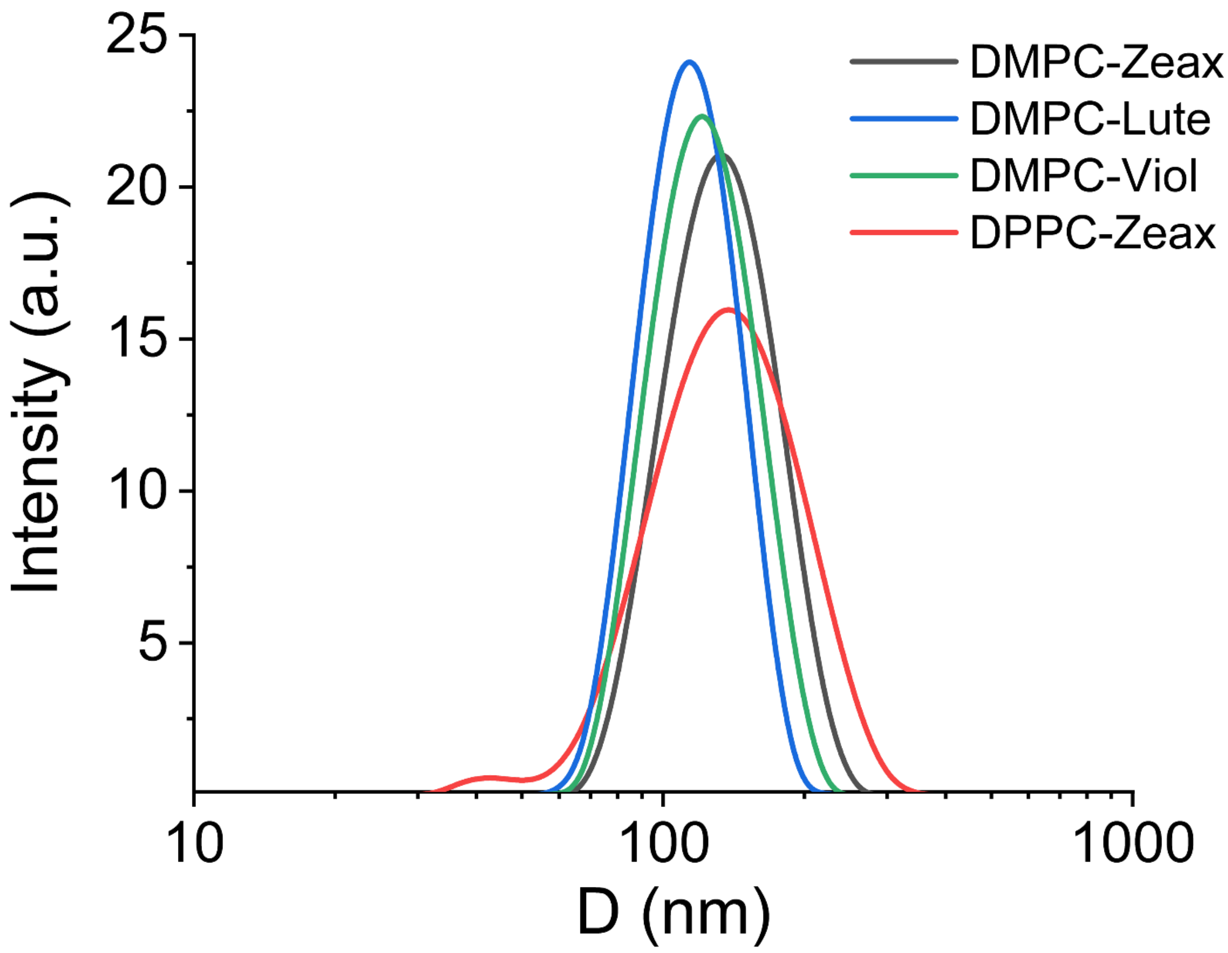

Figure S1. DLS data for liposomes with xanthophylls.

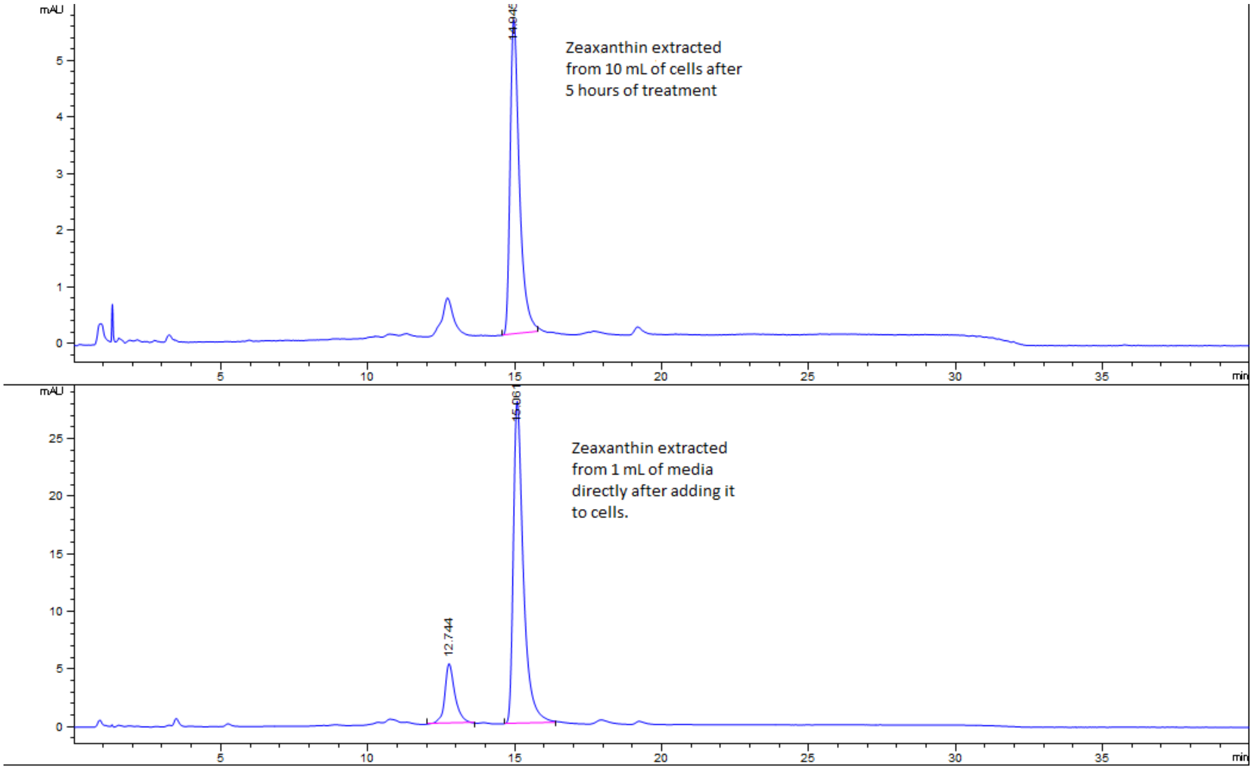

Figure S2. HPLC profile of zeaxanthin extracted from HEK293F cells and media. Zeaxanthin preparation contained residual lutein (zeaxanthin is major peak at 15.1 min, and lutein is peak at 12.7 min). Zeaxanthin was separated with a gradient of 10-60% B, where A=acetonitrile:water:triethylamine (90:10:0.1) and B=100% ethyl acetate [1].

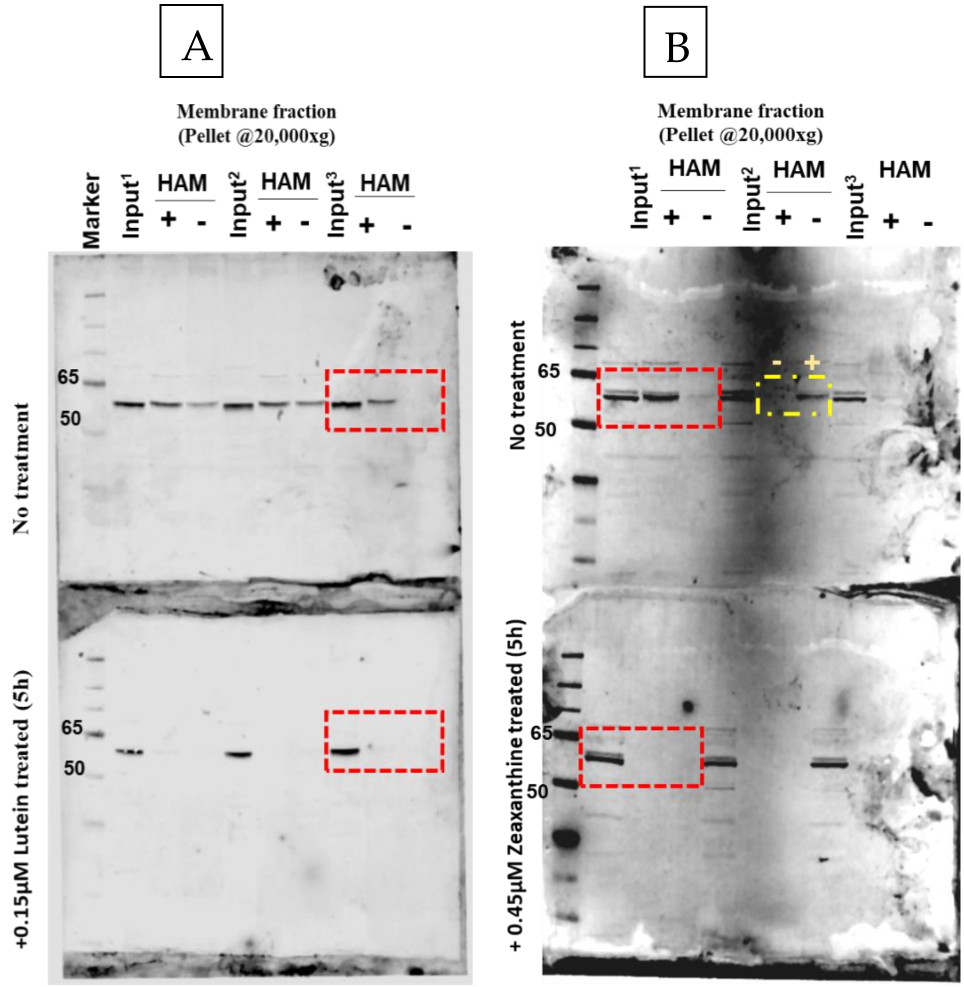
A
Membrane fraction
(Pellet @20,000xg)
Marker
Input1
HAM
+
-
Input2
HAM
+
-
Input3
HAM
+
-
65
50
No treatment
65
50
+0.15μM Lutein treated (5h)
B
Membrane fraction
(Pellet @20,000xg)
Input1
HAM
+
-
Input2
HAM
+
-
Input3
HAM
+
-
65
50
No treatment
- +
65
50
+ 0.45μM Zeaxanthine treated (5h)

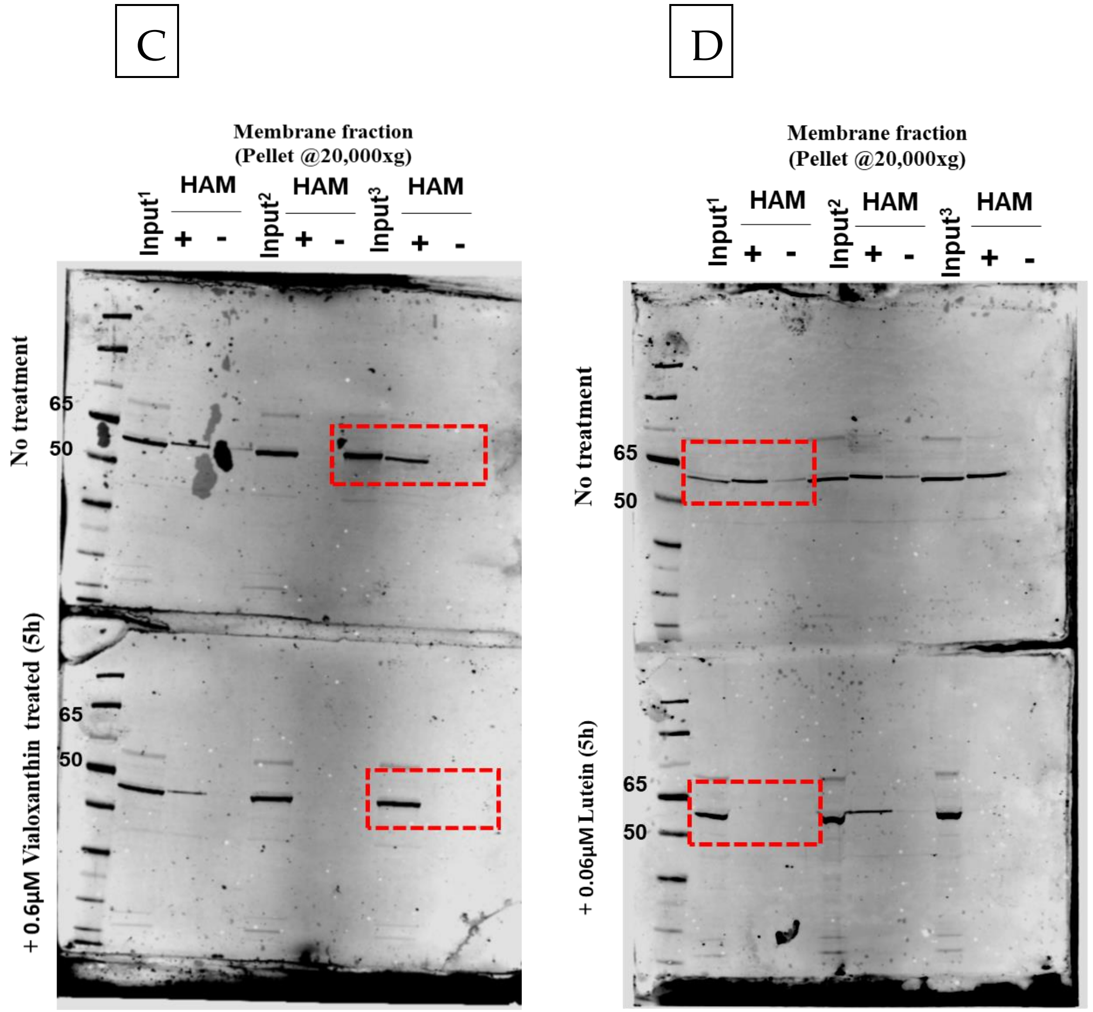

Figure S3. Detection of mouse BCO2 palmitoylation by acyl-RAC assays. Raw western blots.

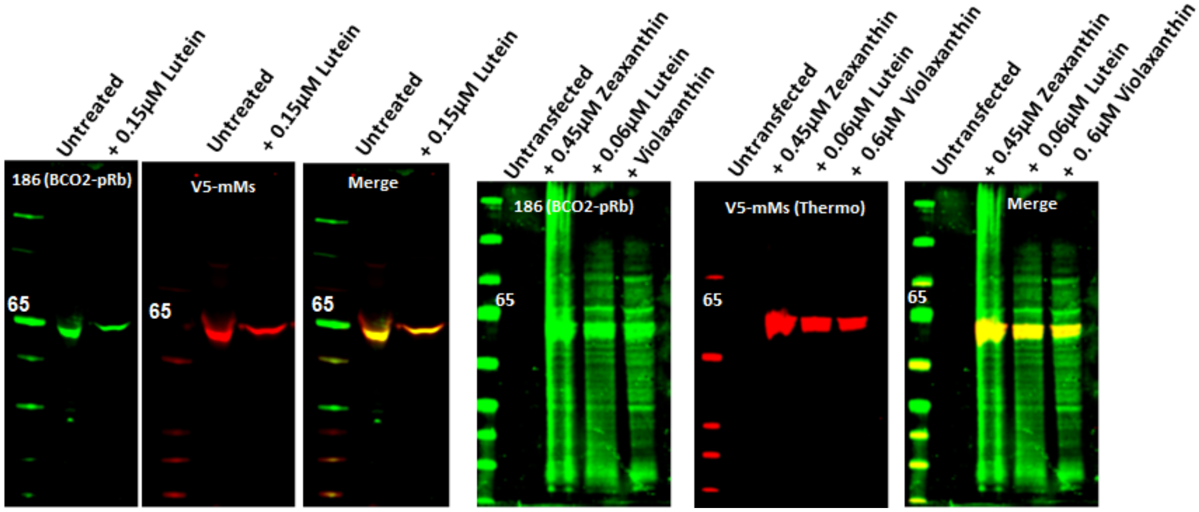

Figure S4. mBCO2 protein expression in COS7 cells transfected with V5-tagged mBCO2. COS7 cells expressing mBCO2 protein treated with different substrates for 5h were grown on poly-L-lysine coated coverslips and subjected to immunolocalization studies using confocal microscopy. For immunoblotting, post-nuclear supernatant was separated by SDS-PAGE. The presence of V5-tagged mBCO2 was probed by immunoblotting with rabbit polyclonal anti-mouse BCO2 (green) and mouse monoclonal anti-V5 (red) antibodies.

1. Cunningham FX, Jr., Gantt E: **A study in scarlet: enzymes of ketocarotenoid biosynthesis in the flowers of Adonis aestivalis**. *Plant J* 2005, **41**(3):478-492.
